# 

**Published:** 1879-08

**Authors:** 


### CONTENTS:

ARTICLE I.—Periostitis—Its Causes and Remedies. By Dr. W. R. Holmes.......... 145
ARTICLE II.—The National Dental Association.......... 153
ARTICLE III.—The Brooklyn Dental Society.......... 160
ARTICLE IV.—American Nervousness—Its Philosophy and Treatment. By Geo. M. Beard, M. D.......... 167
ARTICLE V.—Duplicating Vulcanite Plates. By Dr. W. R. Holmes.......... 174
ARTICLE VI.—On Cysts of the Mouth.......... 178
ARTICLE VII.—Dental Physiology. By Dr. E. Parsons.......... 180
ARTICLE VIII.—The Duties of the Dentist. By Dr. W. W. Ford.......... 183

### EDITORIAL, ETC.

Thimble Blistering as a Vehicle for Morphia.......... 187
Premature Decay of Our Teeth.......... 188

### MONTHLY SUMMARY.

New Sources of Rubber.......... 188

Amalgams in Children's Teeth.......... 189

New Experiments in Anæsthetics.......... 190

Utah Mineral Wax.......... 191

An Anomalous Case of Replantation.......... 191

New Thing for Cleansing Surgical Instruments.......... 192

The Use of Iodoform.......... 192

